# Study on the influence of pore water pressure on shear mechanical properties and fracture surface morphology of sandstone

**DOI:** 10.1038/s41598-024-55834-8

**Published:** 2024-03-08

**Authors:** Jiaxin Cheng, Yixin Liu, Chuanhua Xu, Jiang Xu, Mingzhi Sun

**Affiliations:** 1 State Key Laboratory of Safety and Health for Metal Mine, Maanshan, 243071 China; 2 Sinosteel Maanshan General Institute of Mining Research Co., Ltd, Maanshan, 243071 China; 3 College of Resources and Environmental Engineering, Jiangxi University of Science and Technology, Ganzhou, 342399 China; 4 College of Safety and Environmental Engineering, Shandong University of Science and Technology, Qingdao, 266590 China; 5 State Key Laboratory of Mining Disaster Prevention and Control Co-Founded By Shandong Province and Ministry of Science and Technology, Shandong University of Science and Technology, Qingdao, 266590 China; 6 State Key Laboratory of Coal Mine Disaster Dynamics and Control, Chongqing University, Chongqing, 400044 China

**Keywords:** Variogram, Direct shear test, Pore pressure, Surface morphology, Geophysics, Natural hazards

## Abstract

To further investigate the weakening effect of pore water pressure on intact rock mechanics properties and characteristics of fracture surface after failure, direct shear tests of sandstone were conducted under different pore pressure. A 3D scanner was employed to digitize the morphology of the post-shear fracture surface. The variogram function was applied to quantify the anisotropic characteristics of post-shear fracture surface. The relationship between deformation during shear failure of intact rock and quantitative parameters of fracture surface after shear failure was initially established. It can be found that amplitudes of the sinusoidal surface determine the maximum value of variogram, and period affect lag distance that reach the maximum value of variogram. Test results revealed that the increase of pore pressure has obvious weakening effect on shear strength and deformation of rock. Moreover, the increase of pore pressure makes the shear fracture surface flatter. It can be obtained that both *Sill*_max_ and *Range*_max_ are positively related to shear strain, but negatively related to normal strain.

## Introduction

The development and utilization of underground space and the efficient exploitation of deep resources are important issues affecting the sustainable development of society. The mechanical properties of rock and the characteristics of its fracture surface are very important for understanding the internal processes of the earth, predicting and controlling natural disasters, and reasonably and efficiently exploiting underground resources^1–5^. The study of the rock fracture surface can also improve our ability to prevent and respond to geological disasters, thereby reducing the damage of geological disasters to the environment and communities, and achieving more sustainable community development.

It is well known that these characteristics may be affected by factors such as pore pressure, which in turn affects the strength and strain of the rock, as well as the failure mode of the rock. Alessandro, Verdecchia et al.^6^ analyzed the seismic activity in southern Kansas and found that even fluid diffusion of less than 0.1 MPa would lead to changes in pore pressure and coulomb stress, thus triggering earthquakes. Sun et al.^7^ studied the possibility of triggering earthquakes during the entire hydraulic fracturing process from fracturing to stopping, and considered factors such as fault location, direction and location. Mehrabifard and Eberhardt^8^ found that under the conversion stress state of S_H_ > S_V_ > S_h,_ the reservoir faults with a depth of 1 to 6 km reached the critical slip state more widely under the influence of fluid injection than under the compressive stress state of S_H_ > S_h_ > S_V_, which increased the risk of large earthquakes caused by fluid injection. Hassib et al.^9^ found the basis of fluid injection affecting pore pressure changes and triggering earthquakes at the junction of Abu Dirwa fault, Khor El-Raml area, Seiyal and Kurkur faults. The study of Luo et al.^10^ shows that in the Lijiang-Xiaojinhe fault zone, groundwater penetrates into the deep crust along the fault, which increases the fluid pressure in the pores and accelerates the water–rock chemical reaction, resulting in a decrease in the strength of the fault zone and eventually triggering an earthquake. Dal Zilio et al.^11^ used the H-MEC model to study how the change of pore fluid pressure affects the sliding of seismic faults, and found that fault rupture is related to the porous elastic coupling in the finite width shear zone, and then put forward the importance of considering hydrogeological structure.However, the existing literatures have discussed more about the geological data and monitoring data of the engineering sit^8–10,12–15^, the mechanism of coupled hydromechanical processes are still poorly understood^16–19^ .

It can be obtained by summarizing the aforementioned studies, there are two prerequisites for the failure: (a) rock mass is under compression and shear coupling loading and may be in critical instability state^20^, (b) pore-pressure alteration caused by fluid percolation or injection^21,22^. By constructing high-resolution 3D models of seismic velocity structure and pore pressure field, Tan et al.^23^ concluded that earthquakes are mainly caused by an increase in pore pressure on seismogenic faults due to the presence of water or gas. In addition, the disturbance of pore elastic stress and aseismic slip caused by hydraulic fracturing may also lead to the formation of some earthquake swarms. Wu et al.^24^ proposed that the increase of pore pressure and water wedge effect can lead to two kinds of shear expansion, which occur before the tensile failure. Mei et al.^25^ revealed that under the influence of continuous hydraulic coupling, the specimen showed the characteristics of tensile shear failure. The macroscopic fracture of the outer surface was Z-shaped and the interaction between the cracks increased the number of seepage paths and accelerated the formation of macroscopic cracks.

It is now widely believed that fluids are capable of exerting significant chemical and mechanical influence on earthquake faulting^8^. In laboratory testing, a great deal of works has been done to study the relationship between rock deformation and fluid flow^26–28^. Zhao et al.^29^ found that the fracture network affected by small cracks can lead to significant damage and slip phenomena in rock at the same time. The change of stress will lead to the fluctuation of pore pressure in low permeability rock matrix, which promotes the fracture process of rock. Zhong et al.^30^ conducted an undrained cyclic triaxial compression test on saturated coal samples and found that pore overpressure provided tensile stress, resulting in a large number of macroscopic tensile cracks, which was directly manifested as the expansion and deformation of coal samples. However, direct shear test has obvious advantages in controlling normal load and was often used to study shear mechanism of rock mass^31–33^.

In this paper, laboratory tests which coupling compressive-shear stress and pore pressure were carried out on sandstone samples. The effect of pore water pressure on the degradation of shear properties of intact sandstone is studied. Furthermore, morphologic characteristics of shear fracture surface and anisotropy were analyzed quantitively by variogram. Analyzing the morphology characteristics of the fracture surface can not only infer the fracture process^34,35^, but also further understanding the hydraulic coupling mechanical behavior of rock mass after shear failure^36^.

## Materials and methods

### Sample preparation

**Figure 1:**
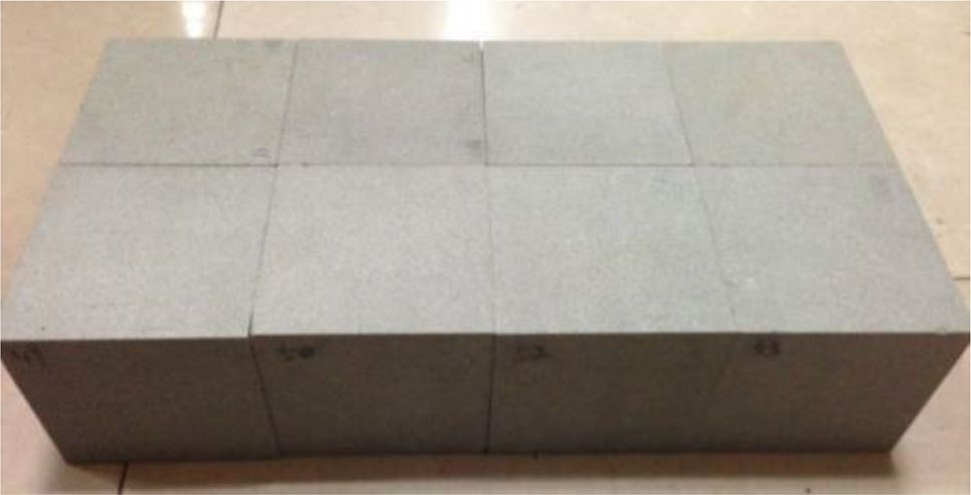
Picture of some sandstone specimen.

Sandstone with better homogeneous isotropy was selected for testing. The sandstones samples were obtained from Three Gorges Reservoir, Chongqing, China. The samples were primarily comprised of quartz, feldspar, chert and muscovite with a grain size distribution of 0.1 to 0.5 mm. Drilling cores without obvious fractures were selected and cut into cubes with dimensions of approximately 100 × 100 × 100 mm^3^. The Young's modulus is 6.79 GPa, the Poisson ratio is 0.26, the uniaxial compressive strength is 81.04 MPa, and the density is 2.32 g/cm^3^. Before testing, the specimen should be saturated. In the saturation process, a dry sample was inundated in a tank filled with purified water for 1 h, and then it was taken out and weighed. The operation was repeated every hour until the weight of the specimen remained unchanged. At this time, the sample was considered to be saturated. Figure 1 shows picture of some sandstone samples.

### Testing apparatus

**Figure 2:**
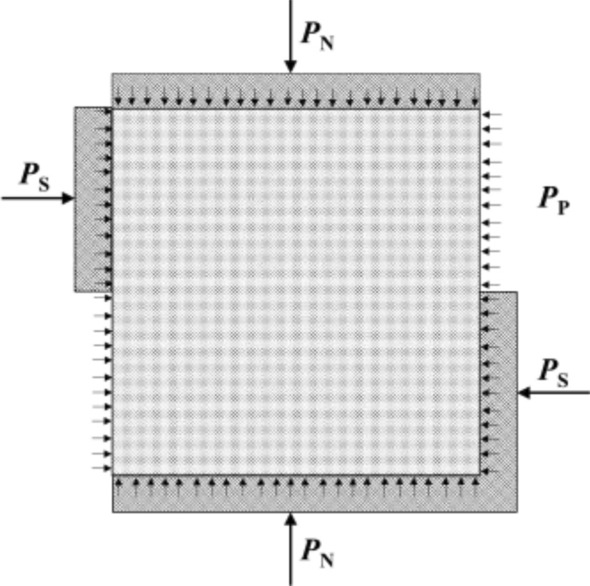
Force diagram of specimens.

A direct shear test apparatus for coupled hydro-mechanical was developed^37^. It was designed to allow for testing rock materials under different loading conditions, including constant load or constant displacement rate. The pore water pressure was loaded by the plunger pump. During the direct shear test, the test system can automatically record the shear force, normal force and displacement data. Force diagram of samples in direct shear test is shown in Fig. 2.The process of direct shear test includes the following two steps: (1) A constant loading rate of 0.05kN/s was applied to the specimen until the predetermined normal stress was reached. (2) through the horizontal actuator, the specimen was loaded at a shear rate of 0.005 mm/s.

**Figure 3:**
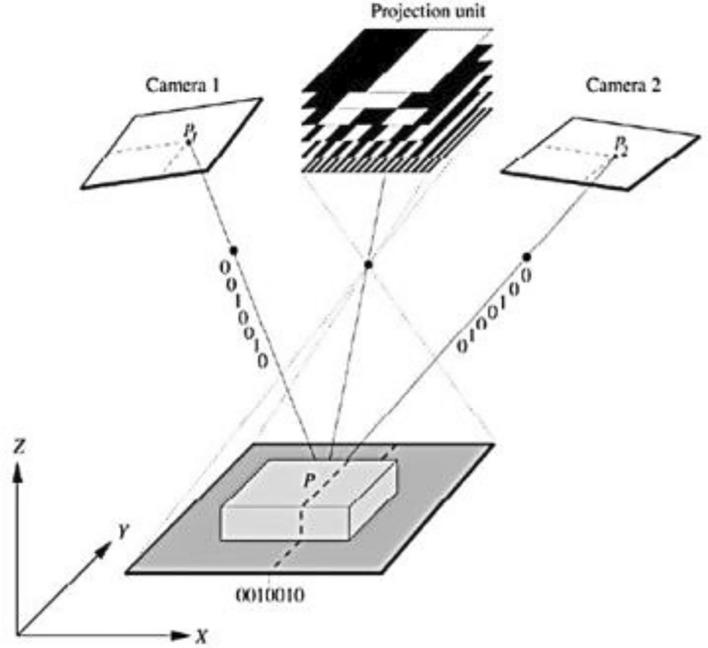
Principle of the advanced topometric sensor system.

The morphology of the shear fracture surface was scanned using an ATS system. The ATS systems provide an optical method based on a combination of white light fringe projection (see Fig. 3), triangulation, and phase shifting for fast and accurate calculation of high-dense 3D point clouds^38^.

### Informed consent

Informed consent was obtained from all subjects involved in the study.

## Results

### Stress and strain

**Figure 4:**
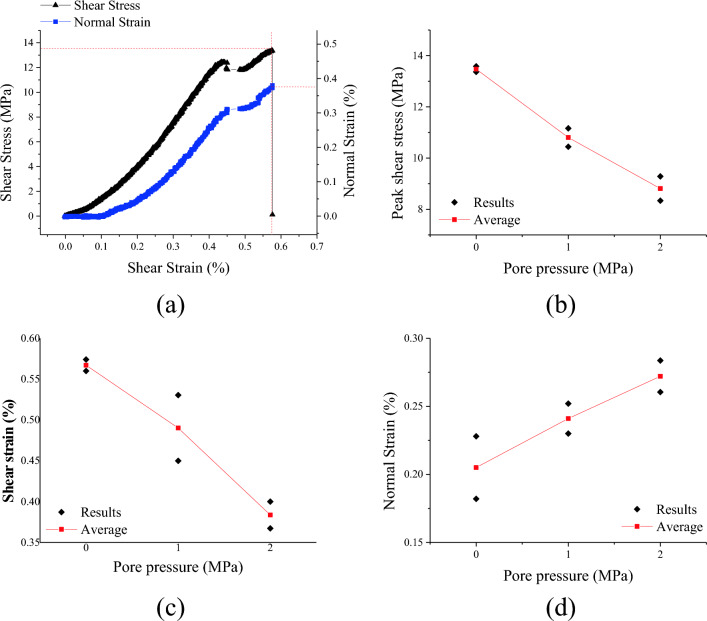
Variation curve of shear failure parameters under different pore pressure, (**a**) shear stress and normal strain versus shear strain with pore pressure = 0 MPa, (**b**) peak shear stress, (**c**) peak shear strain, (**d**) peak normal strain.

Under uniaxial compression conditions, the complete stress–strain curve of a complete rock exhibits an initial stage of upward depression during loading, which is caused by the closure of microcracks inside the rock^39^. Similarly, the shear stress-shear strain also increased in concavity form (see Fig. 4a). The variation trend of shear stress-shear strain curve was more consistent under different pore pressure. Hence, only the parameters at the peak shear stress were compared and analyzed. As shown in Fig. 4b, the peak shear stress decreased with the increased of pore water pressure, which indicated that pore water pressure decreases the effective stress and lowered the friction coefficient between particles and cementitious materials^8^. It was worth noting that with the increase of pore water pressure, the dispersion of peak shear stress increased. This was due to the fact that under compressive-shear stress, tension cracks initiated and propagated first, then were coalesced by shear cracks and leading to shear failure^37,40^. Pore water pressure have a weakening effect that was attributed to a drop in the surface energy which needed to create a new crack^41^. Due to the uncertainty of crack initiation, the influence of water wedge effect different. As pore pressure increased, normal strain increased and shear strain decreased at peak shear stress (see Fig. 4c,d). It indicated that water pressure plays a dominating role in the promotion of tension cracks propagation. In the shear failure process of samples under compressive-shear stress, the normal restraint is less, tension cracks account for a larger proportion, shear cracks account for a smaller proportion, resulting in the decreased of shear strain and the increased of normal strain.

### Morphological characteristics of shear fracture surface

Strength, deformability and flow properties of rock mass depend very much on the surface roughness of joints^42–44^. Usually, roughness on natural rock joint planes is anisotropic. Therefore, it is imperative to know the variation of surface morphology in various directions^45^. For this purpose, the variogram function was employed to identify the roughness characteristics of the surface because it was designed to characterize spatial variations^46^.

The variogram *γ* (*h*, *θ*) is defined as the average of the squares of the delayed amplitude differences:

1 $$\gamma \left( {h,\theta } \right) = \frac{1}{2N\left( h \right)}\sum\limits_{i = 1}^{N\left( h \right)} {\left\{ {Z\left( {x_{i} ,y_{i} } \right) - Z\left[ {\left( {x_{i} ,y_{i} } \right) - h\left( \theta \right)} \right]} \right\}}^{2}$$

where *N*(*h*) is the number of pairs of data whose lag is *h,Z*(*x*_*i*_, *y*_*i*_) is the height at point (*x*_*i*_, *y*_*i*_) and [*Z*(*x*_*i*_, *y*_*i*_) − *h*(*θ*)] is the height at a radial distance *h* in a direction *θ* from (*x*_*i*_, *y*_*i*_).

**Figure 5:**
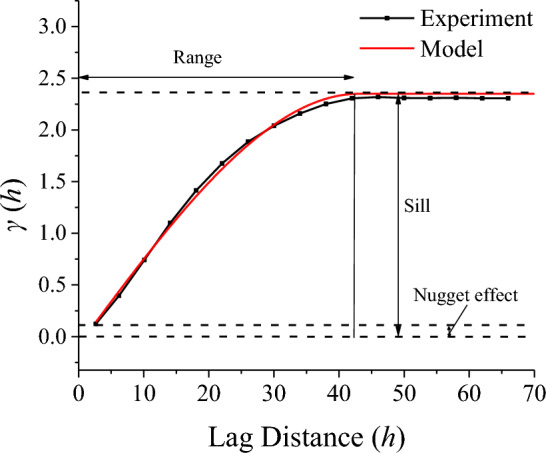
Experimental variograms and best fit spherical model for the fracture surface.

The theoretical variogram models without a sill include the power model and the logarithmic model. The transition models comprise the spherical, exponential and Gaussian models^47^. Here, Spherical model was a used to fit variogram function (see Fig. 5) and defined to be:

2 $$\gamma \left( h \right) = \left\{ {\begin{array}{*{20}c} {0,} & {\left( {h = 0} \right)} \\ {C_{0} + C\left( {\frac{3}{2}\frac{h}{a} - \frac{1}{2}\left( \frac{h}{a} \right)^{3} } \right),} & {\left( {0 < h \le a} \right)} \\ {C_{0} + C,} & {\left( {h > a} \right)} \\ \end{array} } \right.$$

where *C*_0_ is nugget effect, *C* is sill, *a* is range, *h* is lag distance.

**Figure 6:**
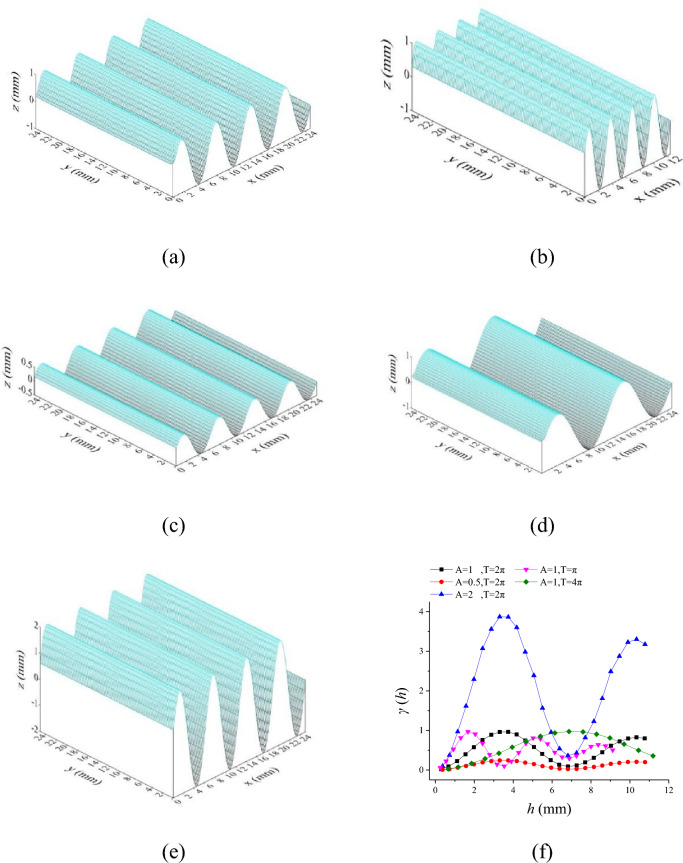
Sinusoidal surface and its corresponding variogram, (**a**) A = 1, T = 2π, (**b**) A = 1, T = π, (**c**) A = 0.5, T = 2π, (**d**) A = 1, T = 4π, (**e**) A = 2, T = 2π, (**f**) variogram.

Figure 6 shows sinusoidal surface with different amplitudes (A) and periods (T) and its corresponding variogram. It can be found that amplitudes (A) of the sinusoidal surface determines the maximum value of variogram (sill), and periods (T) affect lag distance (range) that reach the maximum value of variogram. Therefore, the parameters of the variogram used in this paper include the range *a*, the sill *C*.

**Figure 7:**
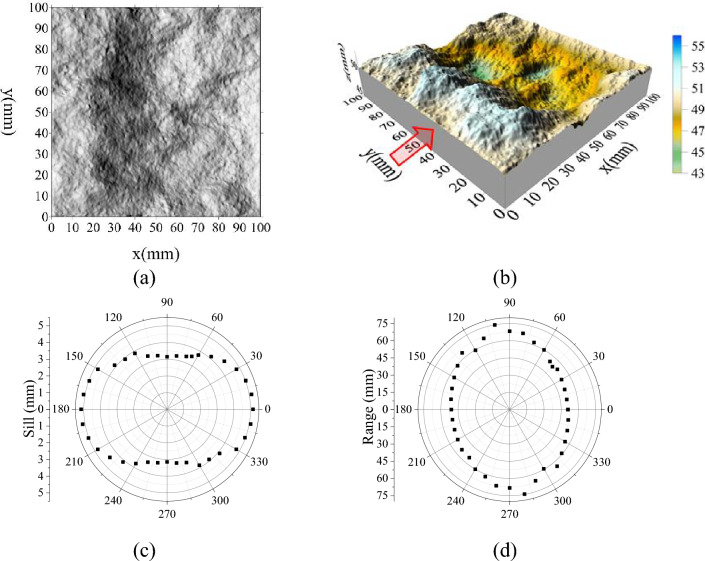
Parameter distribution curve of variogram under pore pressure = 0 MPa, (**a**) contour map, (**b**) 3D map, (**c**) sill, (**d**) range (‘’ represent the direction along the x-axis and the position of the load surface).

Figure 7 shows parameters distribution curve of variogram under pore pressure of 0 MPa. It can be seen that the fluctuation of the fracture surface has obvious directivity, undulating along the shear direction, but it is relatively flat along the vertical direction of shear (see Fig. 7a,b). Correspondingly, the maximum of sill is along the shear direction, and the maximum of range is perpendicular to the shear direction (see Fig. 7c,d). This consistent with the conclusions obtained in Fig. 6. Therefore, it is possible to prove the effectiveness of the sill represents the height of the fluctuation body in fracture surface, and the range represents the single fluctuation body and can reflect frequency of fluctuation.

### Anisotropic evolution under different pore pressure

To quantify the anisotropy of shear fracture surface, the anisotropic coefficient (*ξ*) which defined as the ratio of physical and mechanical parameters in the weakest direction to those in the strongest direction was adopted. The anisotropic coefficient *ξ* was defined as

3 $$\xi = \frac{{\min \left\{ {P_{\theta } } \right\}}}{{\max \left\{ {P_{\theta } } \right\}}}$$

where min{*P*_*θ*_} represented and max{*P*_*θ*_} is the minimum value and the maximum value of the related parameters, respectively.

**Figure 8:**
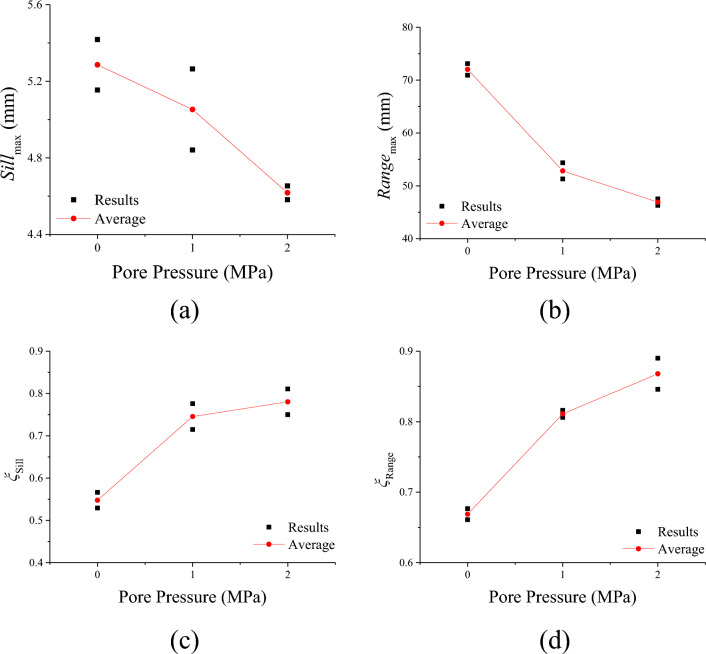
Curves of parameters of variogram function of shear fracture surface under different water content, (**a**) Sill_max_, (**b**) Range_max_, (**c**) *ξ*_sill_, (**d**) *ξ*_range_.

Figure 8 shows curves of parameters of variogram function of shear fracture surface under different pore pressure. It can be seen that as pore pressure increased, the max of sill (*Sill*_max_) and the max of range (*Range*_max_)^48,49^ both decreased. However, the anisotropic coefficient of sill (*ξ*_*Sill*_) and the anisotropic coefficient of range (*ξ*_*Range*_) increased with pore pressure increased. This can be indicated that the fluctuation height of the shear fracture surface reduced and the frequency of fluctuation increased. As mentioned above, pore pressure gradient field can have a dominating effect on the propagation direction of the main tensile crack. Increasing the pore pressure enhanced the number of tension cracks and make the cracking path more complexity. Furthermore, the existence of pore pressure reduced the effective normal stress, the direction of the resultant force tends to the direction of shear. Altogether, the increase of pore pressure makes the shear fracture surface flatter.

## Discussion

It has long been recognized that the roughness of rock discontinuities has a significant impact on the shear strength characteristics of discontinuous rock masses^50^. However, the surface roughness is also a key point which reflects the physics and mechanics of the fracture process^51^. The shear strain and normal strain under compressive-shear stress determined the combination mode of crack propagation^52^. And crack propagation path determined the roughness of fracture surface. Here, the average normal strain and shear strain at peak shear stress and corresponding quantitative parameters of shear fracture surface under different pore pressures were listed (see Table 1). It can be obtained that both *Sill*_max_ and *Range*_max_ are positively related to shear strain, but negatively related to normal strain. The nonlinear fitting function obtained is as follows:

4 $$Sill_{\max } = 6.614 + 2.81\frac{\log (\delta )}{{\nu^{0.411} }}$$

5 $$Range_{\max } = 45.22 + 0.417\left( {\frac{\delta }{\nu }} \right)^{4.092}$$

where *δ* is shear strain at peak shear stress, *ν* is normal strain at peak shear stress.

**Table 1:** Deformation and corresponding quantitative parameters of shear fracture surface under different pore pressures.

Pore pressure (MPa)	Shear strain (10^−2^)	Normal strain (10^−2^)	*Sill*_max_ (mm)	*Range*_max_ (mm)
0	0.567	0.205	5.28599	72.02864
1	0.49017	0.24102	5.05255	52.83605
2	0.38359	0.27208	4.61751	46.91837

By establishing the relationship between strain and fracture surface morphology of intact rock, it is helpful to estimate the second sliding instability of intact rock after shear failure.( The second sliding instability of rock refers to that the rock structure will gradually damage after a period of stable creep under the action of continuous stress, resulting in the development of microcracks and pores in the rock. When these defects reach a certain degree, the creep rate of the rock will suddenly increase, which is the second sliding instability. )

## Conclusions

Coupled hydromechanical processes play a major role in natural crustal processes. It has proved that the pore water pressure has a direct weakening effect on the mechanical properties of rock mass. As the important information, the post-shear fracture surface could not only reflect the process of intact rock failure but also contributes to the second failure of jointed rock. Then, Laboratory tests which coupling compressive-shear stress and pore pressure were carried out on intact sandstone samples. The morphology of the post-shear fracture surface was digitized by using a 3D scanner. The variogram function was employed to quantify roughness of post-shear fracture surface under different pore pressure. The following conclusions were obtained:

1. The increase of pore water pressure plays a dominating role in the promotion of tension cracks propagation and significantly weakens the shear strength of intact rock. The pore water pressure reduces the effective stress which influence crack mode and crack path. When rock sample reach the peak shear stress, normal strain increase and shear strain increase with pore pressure increase.
2. The variogram function is validate to quantify the morphology of fracture surface. The parameters of variogram, sill and range, could reflect the height of the fluctuation body in fracture surface and the frequency of fluctuation, respectively.
3. The shear fracture surface is anisotropic, the change is most obvious along the shear direction with the change of pore water pressure. As pore pressure increased, the max of sill (*Sill*_max_) and the max of range (*Range*_max_) both decreased.
4. The relationship between deformation during shear failure of intact rock and quantitative parameters of fracture surface after shear failure is established. It can be obtained that both *Sill*_max_ and *Range*_max_ are positively related to shear strain, but negatively related to normal strain.

Exploring the mechanism of pore water pressure through the anisotropic characteristics of shear crack surfaces can help to more accurately evaluate the rock mechanical behavior under different groundwater conditions, accurately quantify the surface characteristics of rock cracks, and have important application significance for geological engineering practice, disaster prevention and reduction strategy formulation, and efficient and sustainable development of underground resources.

In this study, we focused on the performance of intact sandstone under the coupling effect of compressive shear stress and pore pressure, but did not take into account the common characteristics of primary fractures in natural rocks. This indicates the limitations of studying rock types and their mechanical states, and also points out the direction of our future research. In addition, this experiment only considers pore water pressure as a single variable and does not involve other potential influencing factors in the natural environment, such as climate change and chemical erosion. In future research, we plan to introduce these additional variables to comprehensively examine the coupling effect of compressive shear stress and pore pressure under the combined action of comprehensive factors.

## Data Availability

All data related to this paper may be requested from the corresponding author.

## References

[1] Zhao Y, Feng Z (2023). A brief introduction to disaster rock mass mechanics. Geohazard Mech..

[2] Miao K (2023). Utilization of broken rock in shallow gobs for mitigating mining-induced water inrush disaster risks and environmental damage: Experimental study and permeability model. Sci. Total Environ..

[3] Wang Z (2024). Dynamic mechanical properties and failure characteristics of layered composite rock containing a tunnel-shaped hole. Theor. Appl. Fract. Mech..

[4] Chen Y, Xu J, Peng S, Zhang Q, Chen C (2023). Space–time evolution law of progressive failure area and mechanical behaviour of rock under different seepage conditions. Eng. Geol..

[5] Li P, Luo N, Suo Y, Zhai C, Sun W (2023). Research on petrophysical properties and porosity evolution of fractured coal mass under cyclic impact for coalbed methane exploitation. Geomech. Energy Environ..

[6] Verdecchia, A., Cochran, E. S. & Harrington, R. M. Fluid-earthquake and earthquake-earthquake interactions in Southern Kansas, USA. *J. Geophys. Res. Solid Earth.* **126** (2021).

[7] Sun Z (2023). Implications for fault reactivation and seismicity induced by hydraulic fracturing. Petrol. Sci..

[8] Mehrabifard A, Eberhardt E (2023). Reliability of earthquake-size distribution and stress regime relationships for fluid-injection-induced seismicity. Geoenergy Sci. Eng..

[9] Hassib GH, Saadalla H, Mohamed HH (2023). Forty years spatio-temporal seismicity distribution and the evidences of the pore pressure impact on triggering earthquakes at the Northern Part of Lake Nasser, Aswan, Egypt. J. Afr. Earth Sci..

[10] Luo Z (2023). Earthquakes evoked by lower crustal flow: evidence from hot spring geochemistry in Lijiang-Xiaojinhe fault. J. Hydrol..

[11] Dal Zilio L, Hegyi B, Behr W, Gerya T (2022). Hydro-mechanical earthquake cycles in a poro-visco-elasto-plastic fluid-bearing fault structure. Tectonophysics.

[12] Coppola M (2021). Meso- to nano-scale evidence of fluid-assisted co-seismic slip along the normal Mt. Morrone Fault, Italy: implications for earthquake hydrogeochemical precursors. Earth Planet Sci. Lett..

[13] Gasperini L, Polonia A, Çağatay MN (2018). Fluid flow, deformation rates and the submarine record of major earthquakes in the Sea of Marmara, along the North-Anatolian fault system. Deep Sea Res. Part II Top. Stud. Oceanography.

[14] Song X, Lei J, Zou K (2023). The 2022 Luding, Sichuan, China, M 6.8 Earthquake: a fluid-related earthquake?. J Asian Earth Sci..

[15] Duan Q, Yang X, Chen J (2017). Hydraulic properties of a low permeable rupture zone on the Yingxiu-Beichuan Fault activated during the wenchuan earthquake, China: Implications for fluid conduction, fault sealing, and dynamic weakening mechanisms. Tectonophysics.

[16] Yan C, Guo H, Tang Z (2022). Three-dimensional continuous-discrete pore-fracture mixed seepage model and hydro-mechanical coupling model to simulate hydraulic fracturing. J. Petrol Sci. Eng..

[17] Song Y, Cheng H (2024). Opening-dependent phase field model of hydraulic fracture evolution in porous medium under seepage-stress coupling. Theor. Appl. Fract. Mech..

[18] Luo Z, Zhang N, Zhao L, Yao L, Liu F (2018). Seepage-stress coupling mechanism for intersections between hydraulic fractures and natural fractures. J. Petrol. Sci. Eng..

[19] Horn R, Fleige H, Mordhorst A, Dörner J (2023). Structure dependent changes in pore water pressure due to stress application and consequences on the effective stress. Soil Till. Res..

[20] Xin J (2023). Mechanical behaviors of backfill-rock composites: physical shear test and back-analysis. J. Rock Mech. Geotech..

[21] Liu X (2023). Effects of seepage pressure on the mechanical behaviors and microstructure of sandstone. J. Rock Mech. Geotech..

[22] Ito T (2008). Effect of pore pressure gradient on fracture initiation in fluid saturated porous media: Rock. Eng. Fract. Mech..

[23] Tan Y (2023). Tomographic evidences for hydraulic fracturing induced seismicity in the changning shale gas field, Southern Sichuan Basin, China. Earth Planet Sci. Lett..

[24] Wu R (2018). Numerical investigation of fluid injection into poorly consolidated geomaterial considering shear dilation and compaction. J. Petrol. Sci. Eng..

[25] Mei J, Yang L, Zhang W, Rong Y, Zhang X (2023). Stress corrosion and interaction behaviour of adjacent cracks in rock-like material under hydro-mechanical coupling: An experimental and numerical study. Mater Design..

[26] Zhang S (2023). Research on the inherent mechanism of rock mass deformation of oil shale in-situ mining under the condition of thermal-fluid-solid coupling. Energy.

[27] Ma Z, Wang Y, Zheng Y (2022). In situ dynamic X-ray imaging of fluid-rock interactions inside tight sandstone during hydraulic fracturing: fluid flow process and fracture network growth. J. Petrol. Sci. Eng..

[28] Makhnenko RY, Ge C, Labuz JF (2020). Localization of deformation in fluid-saturated sandstone. Int. J. Rock Mech. Min..

[29] Zhao C, Zhang Z, Wang S, Lei Q (2022). Effects of fracture network distribution on excavation-induced coupled responses of pore pressure perturbation and rock mass deformation. Comput. Geotech..

[30] Zhong C, Zhang Z, Ranjith PG, Zhang C, Xue K (2021). The role of pore pressure on the mechanical behavior of coal under undrained cyclic triaxial loading. Rock Mech. Rock Eng..

[31] Zou L, Cvetkovic V (2023). A new approach for predicting direct shear tests on rock fractures. Int. J. Rock Mech. Min..

[32] Vizini VOS, Futai MM (2021). Mode II fracture toughness determination of rock and concrete via modified direct shear test. Eng. Fract. Mech..

[33] Shuai, H., Yingying, G., Xianzhong, L. & Ruitian, Z. Shear stress distribution in rock-cemented discontinuities under direct shear: Theoretical analysis and numerical validation. *Int. J. Geomech.* **22** (2022).

[34] Ma G (2023). Experimental study on acoustic emission and surface morphology characteristics of concrete under different fracture modes. Theor. Appl. Fract. Mech..

[35] Li B (2023). Approach to characterize rock fracture surface: Insight from roughness and fractal dimension. Eng. Geol..

[36] Ji J (2023). Study on the effect of fracture morphology on fracture deformation based on the thermal-hydraulic-chemical-deformation coupling model. Energy.

[37] Yixin L, Jiang X, Shoujian P (2016). An Experimental investigation of the risk of triggering geological disasters by injection under shear stress. Sci. Rep.-UK.

[38] Babanouri N, Nasab SK (2015). Modeling spatial structure of rock fracture surfaces before and after shear test: A method for estimating morphology of damaged zones. Rock Mech. Rock Eng..

[39] 李建林. *岩石力学* [318]重庆大学出版社

[40] Muñoz-Montecinos J, Angiboust S, Garcia-Casco A, Glodny J, Bebout G (2021). Episodic hydrofracturing and large-scale flushing along deep subduction interfaces: implications for fluid transfer and carbon recycling (Zagros Orogen, Southeastern Iran). Chem. Geol..

[41] Ding S, Tang S, Jia H, Li Y (2023). The influence of water on the failure characteristics of sandstone under uniaxial compression conditions by acoustic emission and NMR observation. Eng. Geol..

[42] Kulatilake PHSW, Balasingam P, Park J, Morgan R (2006). Natural rock joint roughness quantification through fractal techniques. Geotech. Geol. Eng..

[43] Cen D, Huang D, Ren F (2017). Shear deformation and strength of the interphase between the soil–rock mixture and the benched bedrock slope surface. Acta Geotech..

[44] Belem T, Souley M, Homand F (2007). Modeling surface roughness degradation of rock joint wall during monotonic and cyclic shearing. Acta Geotech..

[45] Kumar R, Verma AK (2016). Anisotropic shear behavior of rock joint Replicas. Int. J. Rock Mech. Min..

[46] Marache A, Riss J, Gentier S, Chilès JP (2002). Characterization and reconstruction of a rock fracture surface by geostatistics. Int. J. Numer. Anal. Met..

[47] Indraratna B, Thirukumaran S, Brown ET, Premadasa W, Gale W (2014). A Technique for three-dimensional characterisation of asperity deformation on the surface of sheared rock joints. Int. J. Rock Mech. Min..

[48] Gressier J (2010). Control of pore fluid pressure on depth of emplacement of magmatic sills: An experimental approach. Tectonophysics.

[49] Sow D (2017). Modeling the spatial variability of the shear strength of discontinuities of rock masses: application to a dam rock mass. Eng. Geol..

[50] Wang Z (2019). Shear stress relaxation behavior of rock discontinuities with different joint roughness coefficient and stress histories. J. Struct. Geol..

[51] Surface Roughness and the Physical Properties of Fractures: Brown, S R Rock Mechanics Contributions and Challenges: Proc 31St Us Symposium, Golden, 18–20 June 1990P269–276. Publ Rotterdam: A a Balkema, 1990. *International Journal of Rock Mechanics and Mining Sciences & Geomechanics Abstracts*. 28, A215 (1991). 10.1016/0148-9062(91)90732-2.

[52] Chao Y (2021). New crack initiation model for open-flawed rock masses under compression–shear stress. Theor. Appl. Fract. Mech..

